# Multi-parametric evaluation of autologous cultivated Limbal epithelial cell transplantation outcomes of Limbal stem cell deficiency due to chemical burn

**DOI:** 10.1186/s12886-020-01588-6

**Published:** 2020-08-06

**Authors:** Ozlem Barut Selver, Mehmet Gurdal, Ayse Yagci, Sait Egrilmez, Melis Palamar, Turker Cavusoglu, Ali Veral, Cagri Guven, Utku Ates, Zheng Wang, J. Mario Wolosin

**Affiliations:** 1 Departments of Ophthalmology, Ege University Faculty Of Medicine, 35100 Bornova-, Izmir, Turkey; 2 Departments of Medical Biochemistry, Ege University Faculty Of Medicine, Izmir, Turkey; 3 Departments of Histology and Embryology, Ege University Faculty Of Medicine, Izmir, Turkey; 4 Departments of Pathology, Ege University Faculty Of Medicine, Izmir, Turkey; 5 Departments of Gynecology and Obstetric, Ege University Faculty Of Medicine, Izmir, Turkey; 6 Department of Histology and Embryology, Bilim University, Istanbul, Turkey; 7 Department of Ophthalmology and Stem Cell Institute, Icahn School of Medicine at Mount Sinai, Box 1183, One Gustave Levy Pl., New York, NY 10029 USA

**Keywords:** Cultured limbal epithelial cell transplantation, Limbal stem cell deficiency, Ocular surface parameter, Visual function-related quality of life

## Abstract

**Background:**

The sparsity of established tools for the grading of limbal stem cell deficiency hinder objective assessments of the clinical outcome of cultivated limbal epithelial cell transplantation. To advance towards the development of standards for the comparison of the outcomes of these bio-surgical protocols we have now applied a battery of recognized objective and patient-declared subjective outcome criteria to the autologous modality of cultivated limbal epithelial cell transplantation.

**Methods:**

The prospective study involved ten patients (M/F = 9/1; mean age = 42.1 years) displaying overt unilateral limbal stem cell deficiency complying with the inclusion criteria described in Methods. Limbal biopsies were obtained from the contralateral eye and their outgrowths after 2-week cultures were transplanted on the affected eye after pannus resection. Outcomes were followed up for 12 months. The objective tests were scores for best-corrected visual acuity (BCVA); using the LogMAR scale, a multiparametric ocular surface score (OSS), and the Schirmer’s test. Subjective scores were based on patient answers to a) perception of visual improvement/pain; b) the 25-item National Eye Institute Visual Function Questionnaire (NEI-VFQ 25); and c) the 12-item Ocular Surface Disease Index Questionnaire (OSDI). All procedures were performed under good manufacture practices using solely xeno-free reagents. In all cases, a single biopsy was divided into two pieces and they were expanded in order to prevent outgrowth failure. In 5 patients, both biopsies generated healthy culture sheet. In those cases the lesser outgrowth were used for immune-histological characterization.

**Results:**

The experimental parallel outgrowth samples showed a similar percent of p63α^+^ cells. PreOp and 12-month PostOp BCVAs and OSSs were, respectively, 1.15 ± 0.70; 0.21 ± 0.13 and 7.40 ± 2.01; 2,30 ± 1.30, (*p* < 0.05). Patient’s responses to all three question sets except ocular pain were consistent with significant improvement (p < 0.05).

**Conclusion:**

Objective clinical metrics demonstrate that in patients with limbal stem cell deficiency, cultivated limbal epithelial cell transplantation improves vision and ocular surface health and subjective visual perceptions.

## Background

Limbal stem cell deficiency (LSCD) is a condition induced by a number of diverse factors, ranging from genetic abnormalities to accidental limbal injuries. It develops when the epithelial stem and precursor cell systems of the limbal zone undergo numerical or functional degradation, thereby compromising the regenerative capacity of the corneal epithelium [1–5]. Ensuing central corneal epitheliopathy results in reduced visual acuity, chronic inflammation, corneal pain, photophobia, epiphora, and blepharospasm, which when bilateral, may progress to full blindness. A consensus for the classification and grading of LSCD has been recently proposed [6]. Worldwide, the prevalence of LSCD is estimated to be between 1 to 9 cases per 100,000 individuals [7]. For unilateral LSCD the autologous transplantation of culture-expanded limbal epithelial cell (CLET) from these biopsies, represents a promising treatment with success rates of about 70–80% [8–17]. For the more critical bilateral LSCD in spite of the hindrance presented by potential immune-rejection, allografts from semi-compatible donors are being implemented [18, 19]. To date, no specific international consensus on standards for the use of objective clinical parameters has been established. To contribute to the development of such standards, we have now subjected our clinical CLET work, completed under the guidelines for good laboratory practices (GLP) to a battery of recognized objective and patient-declared subjective outcome criteria.

## Methods

### Clinical metrics

Best-corrected visual acuity (BCVA) was determined using a standard printed Snellen eye chart with the patient 6 m away. Results are presented as the logarithm of the minimum angle of resolution (LogMAR) scale; the 0 and 1 LogMAR values correspond to 20/20 and 20/200 BCVAs in the popularly known Snellen scale, respectively. Slit-lamp photos were obtained from all patients during each pre and postoperative visit. Ocular surface score (OSSs) were calculated using the Clinical Outcome Assessment in Surgical Trials (COASTL) assessment tool [19]. We used the sum of scores for four parameters, corneal epithelial haze, superficial corneal neovascularization, corneal epithelial irregularity, and corneal epithelial defect. Each parameter was graded between 0 and 3, thus rendering OSS values from 0 (best) to 12 (worst). All OSS parameter determinations were performed by a single researcher (OBS) according to the slit lamp examination and anterior segment photography. For the Schirmer’s test without topical anesthesia, we measured the advance of fluid on the filter paper 5 min after insertion of the filter paper into the conjunctiva. Recommended cutting values are 5 and 10 mm [20]. Subject evaluation of changes in the extent of visual loss in the affected eye and in ocular pain was graded separately by using a 0–10 scale in response to the question “Using the endpoints that are marked as guides, how would you rate overall diseased eye’s visual quality / pain?”. In these grading scales, for subjective visual loss zero means perfect vision and for ocular pain zero means no pain.

Finally, we used two wide scope questionnaires for subjective patient evaluation of outcomes. The Ocular Surface Disease Index (OSDI) [21] is a 12- question set that provides a quick evaluation of the ocular discomfort symptoms according to dry eye and their effect on vision-related functioning in the past 2 weeks. It includes assesses the severity of symptoms, functional problems, and environmental factors. Each answer is scored on a 5-point scale, resulting in a total OSDI score ranging from 0 (no symptoms) to 100 (maximum symptoms). OSDI The Turkish version of OSDI [22] was applied in order to obtain OSDI scores. OSDI was applied preoperatively and postoperative 1st, 3rd, 6th, 12th month. At The 25-Item National Eye Institute Visual Function Questionnaire (NEI-VFQ-25, part-1, questions regarding general health; part-2 questions, related to difficulty with activities and quality of life and part-3 questions related to vision, was applied with a modification for Turkish medical practice at preoperatively and postoperative 12th month [23]. In parts 1 and 2 (questions 1 to 16) best to worse condition is graded on a 1 to 5 or 6 scales, yielding ranges of between 4 and 21 and 9 and 455, respectively. In part 3 the 9 questions (17 to 25), though, gradation is on a 1 to 5 worse to best scale, establishing a 9–45 range.

### Statistics

Friedman and Wilcoxon’s tests were used to generate a probability (p) value, where *p* < 0.05 is taken as statistically significant.

### Patient recruitment

Inclusion criteria were; unilateral LSCD due to an ocular chemical burn, a minimum duration after injury of 12 months with best corrected visual acuity (BCVA) equal or worse than 0.4 LogMAR units and Ocular surface score (OSS) equal or exceeding 5 out of 12. Exclusion criteria were; the concomitant existence of any systemic diseases or other ocular conditions and pregnancy or lactation. The study was approved by the University Research Ethics Committee and the Turkish Ministry of Health. The experimental sample comprised nine males and one female, mean age 40.1 representing all needed cases at the Ege University School of Medicine, Ophthalmology Department between 01/10/2015 and 01/09/2017. The mean period between chemical injury and surgical intervention was 121 months (range, 12–504 months).

### Human amniotic membrane (hAM) collection and processing

Human placentas were obtained under sterile conditions from planned, uneventful cesarean sections. Immediately after surgery, under sterile conditions, the hAM was bluntly dissected from the chorion, rinsed with buffered saline containing penicillin, streptomycin, and amphotericin B. Square hAM sections (2.5 × 2.5 cm) were placed epithelial side up into 35 mm Petri dishes containing a Dulbecco Modified Minimal Essential Medium (DMEM): glycerol 1:1 solution. The dishes were transferred to a − 80 °C freezer and subsequently to liquid nitrogen for long term storage. Thawing was performed by letting the hAM rest at room temperature until the substance became liquid. To remove the hAM epithelium and expose the basal lamina, hAMs were incubated in for 1 h at 37 °C in ethylenediaminetetraacetic acid, pH 7.4, followed by light scraping with a dull steel tool. To ensure the absence of transmissible infectious agents in the hAM, blood samples collected from the placental donor, following written consent, 2 weeks before parturition and 6 months afterward and prior to hAM utilization, were serologically examined for HIV-1/2, hepatitis B, hepatitis C and, syphilis. Autologous serum was generated from 10 ml of blood extracted by phlebotomy from the antecubital fossa of the transplant recipient. After spontaneous coagulation, cells were spun down and the serum supernatant was passed through a 0.2 μm filter.

### Limbal tissue collection and culture

A small corneal-limbal biopsy (2 × 1.5 mm, approximately 100 μm thick) was taken from the contralateral healthy eye of the patient at 0°, with care to exclude conjunctival tissue and immediately transported to the cell culture facility in corneal storage medium. Biopsy tissues were divided in to two parts and each part placed epithelial-side up at the center of a 2 cm- diameter preserved human amniotic membrane (hAM) and overlaid with 1.7 ml of epithelial medium consisting of a 1:1 mix DMEM and Ham F-12 (D/F12) complemented with 20% autologous serum and 1% PS. Explants were examined daily and fed with 2 ml of the medium on alternate days. In all cases (at least one outgrowth) an epithelial outgrowth was observed after 3–4 days and it was allowed to expand for between 12 and 14 days, to reach full hAM coverage. In 5 cases, both culture outgrowths were expanded. These parallel five culture tissues were used to evaluate the growth potential of the cultivated outgrowth by determining the percent of basal cells that are overtly positive for p63α, the nuclear protein that marks the extended proliferative potential of ectodermal-derive stratified epithelial cells [23, 24]. Finally, to protect patients for the implicit risks in the contact with animal-derived components or poorly monitored culture environments, all our procedures adhered to the GLP guidelines [25].

### Histology and immunochemistry

Cultures were fixed in formalin for 48–72 h embedded in paraffin and sectioned. Sections were alternative stained with H&E or immune-stained. Sections within 1 mm of the explant were permeabilized with 0.5% Triton X-100 in PBS for 30 min, blocked with 1% BSA-PBS for 2 h and incubated for 18 h at 4 °C with a rabbit monoclonal antibody against p63α (ThermoFisher, Fair Lawn, NJ), After three PBS washes, the sections were incubated with an Alexa 488-conjugated goat anti-rabbit antibody for 30 min and, following 3 PBS washes in PBS- 1% Tween 20, the sections were mounted in DAPI complement VectaShield (Vector Labs. CA). The Alexa 488 and DAPI fluorescent emissions were captured in an epifluorescent microscope and the percent of p63α-positive cells were determined from counts that included at least 100 basal cell nuclei.

### Transplantation surgery

After conjunctival peritomy with Vannas scissors, pannus tissue was removed by blunt dissection to expose the underlying corneal stroma and a superficial keratectomy was achieved by polishing the affected areas with a diamond burr. Occasional bleeding was arrested by topical cautery of the episcleral vessels. The epithelium-covered hAM was then affixed over the prepared stroma by 8 symmetrically spaced conjunctival 10.0 nylon sutures. A second protective, freshly thawed-out hAM was secured by interrupted 8.0 vicryl sutures as a dressing over the transplanted epithelia-hAM sheet. All surgical procedures were performed by a single surgeon (AY).

### Post-operative management

Patients were treated daily with a combination of preservative-free drops of moxifloxacin (0.5%) (Vigamox®, Alcon, USA), 1% loteprednol etabonate (Lotemax®, Bausch &Lomb, U.K) and artificial tears. Moxifloxacin was stopped 10 days after the surgery and loteprednol was tapered slowly in 3 months according to the severity of ocular surface inflammation. Once the overlaying hAM waned away cyclosporine eye drops (Restasis®, Allergan, USA) were added to reduce the risk of dry eye-related inflammation. Topical artificial tear and cyclosporine treatment were continued for the duration of the study. Donor and recipient eyes were examined after 1, 3, 5, 7, 14 and 30 days and 2, 3, 6 and 12 months. Extra visits were performed in case of clinical need. Bio-microscopic anterior segment photographs were taken at each visit. The parametric test values described below were collected Preop and at the 1, 3, 6 and 12-month Postop visits.

## Results

**Fig. 1:**
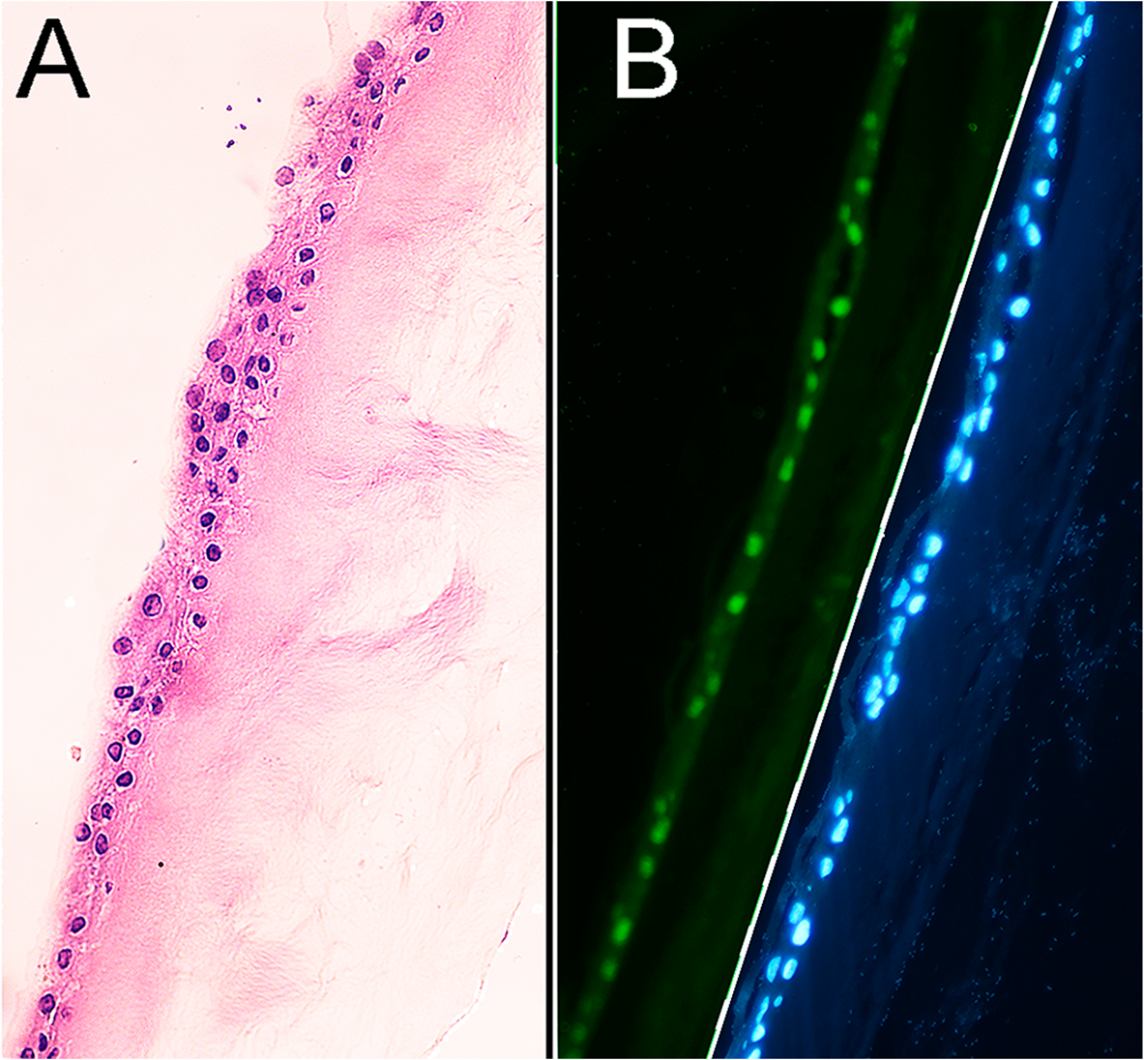
Immuno-histology of the limbal explant outgrowth. **a**. H&E stain. **b**. p63α (left side) and DAPI (right side) stains. Note that the immunogen is present exclusively on basal cells

**Fig. 2:**
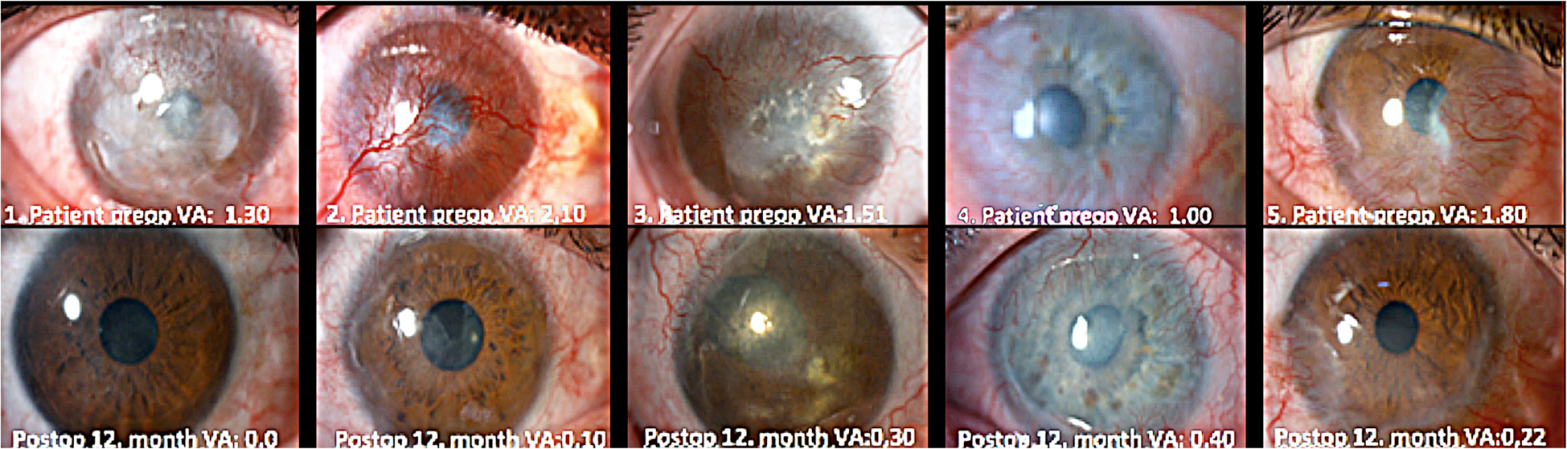
Preop and 12-months Postop ocular surface micrographs and respective BCVA values. VA = BCVA

All contralateral limbal donor eyes showed no change in visual acuity during the follow-up period or resulted in reports of pain or discomfort by the subjects. Figure 1 depicts representative results of the characterization of the six successful, in-parallel cultures that were not used for transplantation. Figure 1, a shows an H&E staining in the proximity of the biopsy. The epithelium consists of 2–3 layers of cells. There were no visible flattened cell profiles typical of the stratified cell when the epithelium is at rest. Figure 1, B shows an immunofluorescence micrograph of p63α (green) stain side-by-side with its corresponding nuclear DAPI blue counterstain. The percent of p63α-positive cells within the basal cell layer was high and quite similar for all six explants. The highest and lowest percentiles were, 56 and 47%, respectively. A clear feature of the staining pattern was the complete absence of protein staining in suprabasal cells; either p63α-positive cells were resilient to the upward mobility pressures. A second possibility, an extremely rapid down-regulation and degradation upon stratification seem less likely. Figure 2 shows micrographs of pre-operative (Preop) and 12 months post-operative (Postop) corneal surfaces for 5 of the treated subjects. The corresponding BCVAs (VA) are stated in the micrographs.

**Fig. 3:**
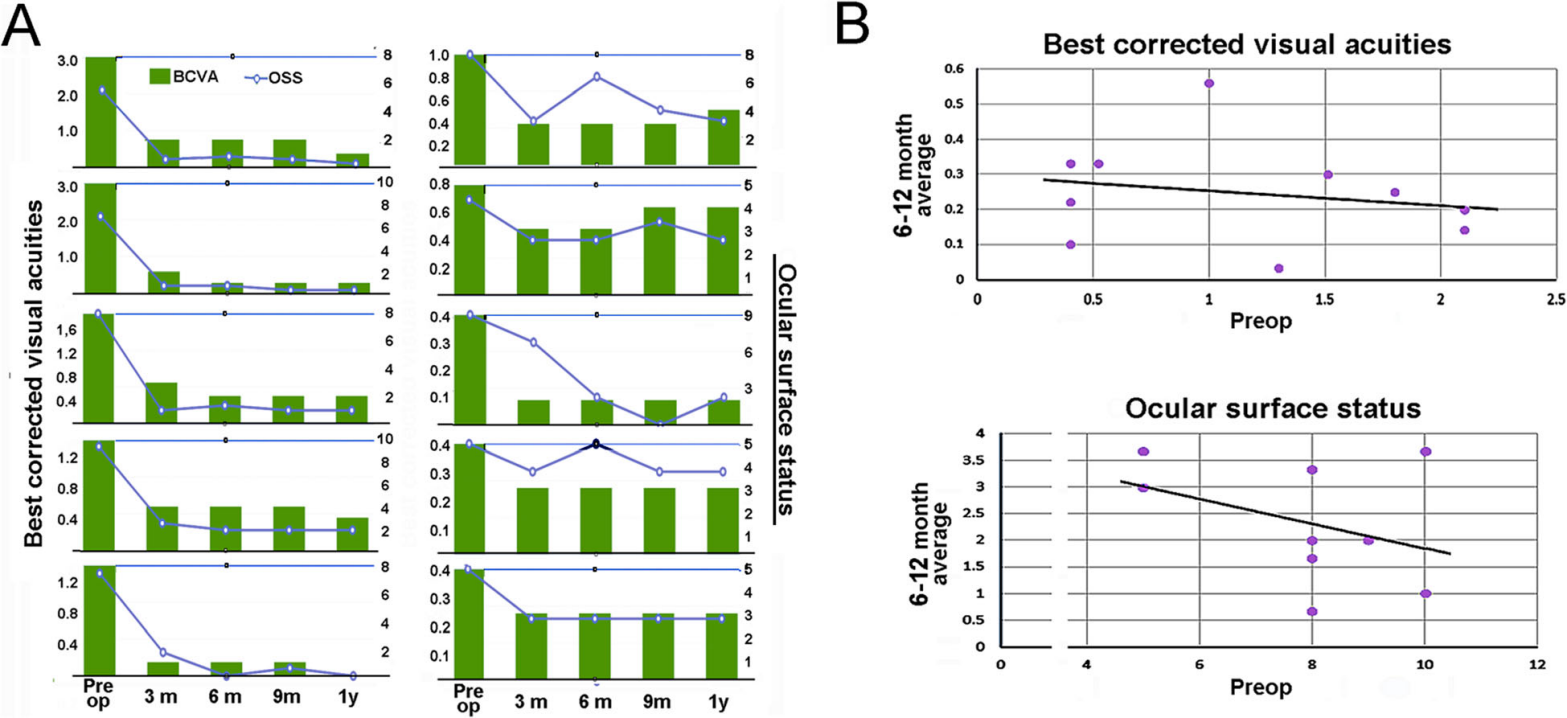
Visual loss and ocular surface scores for all the patients included in this study. **a**. Raw data of ten patients. Overall both parameters follow a coincident trend. **b**. Scatter plots of the Preop score and the average for the scores obtained at 3, 6 and 12 months. The regression lines are shown

Table 1 displays the score ranges and average ± SD scores for all six criteria used. Pre- (control), and postoperative mean best-corrected visual acuity at 3, 6 and12-month were 1.15 ± 0.70; 0.28 ± 0.07; 0.29 ± 0.21 and 0.21 ± 0.13 LogMAR, respectively (*n* = 10; *p* < 0.05 vs all other times). Corresponding mean OSSs were 7.40 ± 2.01; 2.60 ± 0.84; 2.40 ± 0.96; 2,50 ± 1.08, (p < 0.05 vs. control). Pre-, and 1st year post-operative mean subjective visual losses were 7.30 ± 1.49 and 3.50 ± 1.92, respectively (*p* = 0.018). Patients’ responses to all parts of the 25-Item National Eye Institute Visual Function Questionnaire (Turkish version) were consistent with significant visual improvement (p < 0.05). Ocular pain score had no significant difference. In brief, objective visual criteria, BCVA and OSS, were significantly improved from the first determination after 1 month onward and continued to improve over the next 2 months. Three months after the surgery the LogMAR has undergone a 75% decrease (corresponding to a change from 20/290 to 20/35 in the Snellen scale) with a corresponding decrease of 60% in OSS. Notably, there were no further statistically significant improvements over the next 9 months. Lacrimation as measured by the Schirmer’s test was in the normal range prior to the surgical intervention and was not modified by it. Overall, in line with the changes in the objective criteria, the patient’s perceptions of vision and pain decreased by half from by the third Postop month. The scores of the OSDI and first two subparts (P1 and P2) of the NEI-VFQ criteria underwent moderate decreases. The high standard deviation intrinsic to these subjective tests, though, impacted the chances for statistically significant differences at many time points. The changes in two BCVA and OSS, for each individual patient, are shown in Fig. 3 A. and the correlation between the preoperative scores and the average for 3, 6 and 12-month scores is shown in Fig. 3 B for both BCVA (top) and OSS (bottom). These latter panels clearly show that there was no correlation between Preop scores and Postop outcome (*p* > 0.5).

**Table 1:** Average scores and significance relative to the PreOp status for objective and subjective parameters used to evaluate the outcome of treatment of LSCD by autologous CLET

	***Scoring Range***	***PreOp***	***PostOp***				***p-value Preop-Postop month***			
			***1st month***	***3rd month***	***6th month***	***12th month***	***1st***	***3rd***	***6th***	***12th***
	***Objective tests***									
***BCVA***	N-0	1.15 ± 0.70	0.51 ± 0.19	0.28 ± 0.07	0.29 ± 0.21	0.21 ± 0.13	1.000	0.006	0.035	0.000
***OSS***	0–12	7.40 ± 2.01	2.9 ± 0.99	2.60 ± 0.84	2.40 ± 0.96	2,50 ± 1.08	0.107	0.010	0.001	0.000
***Schirmer’s***		21.9 ± 11.6	NM	21.2 ± 8.4	17.7 ± 5.5	18.5 ± 4.7	NM	0.905	0.285	0.369
	***Subjective tests***									
***Vision loss***	0.10	7.30 ± 1.49		3.70 ± 1.56	3.60 ± 1.50	3.50 ± 1.71		0.047	0.019	0.005
***Pain***	0–10	3.60 ± 2.98		1.20 ± 1.03	0.90 ± 1.28	1.80 ± 1.68		0.040	0.050	0.206
***OSDI***	0–100	71.7 ± 26.5	52.97 ± 19.2	53.0 ± 15.4	52.5 ± 20.8	50.9 ± 22.6	0.128	0.181	0.075	0.042
***NEI-VFQ-P1***	4–21	14.70 ± 2.66*				9.50 ± 2.50				0.00
***NEI-VFQ-P2***	13–81	39.90 ± 13.45				25.40 ± 10.37				0.00
***NEI-VFQ-P3***	9–45	20.60 ± 7.29				37.20 ± 7.71				0.00

## Discussion

CLET is an advanced treatment method for LSCD. In autologous transplantations, where systemic immunosuppression and its attendant sequel are avoided, success rates approach 80% [6]. Since its first reports in the late nineties, there have been raising amounts of publications reporting the clinical outcomes of the modality. However, the self-reporting of outcomes in these studies, are usually based on subjective grading, or do not depend on clear criteria, which causes bias of the success dependability. Given the increased frequency of this limbal stem cell transference procedure and its expansion to an increasing number of countries as national regulatory organisms approve its implementation, the development of universally accepted outcome criteria becomes essential to objectively compare the relative advantage of alternative CLET approaches for patient safety, vision improvement, and cost-effectiveness.

Shortt and collaborators have proposed and exemplified a scoring system for evaluating the LSCD inter-, and intra-observer agreements to evaluate the outcome of allogeneic CLET treatment for Steven Johnson Syndrome-induced LSCD [19]. In the present study, to evaluate the outcomes of autologous tissue-based CLET interventions, we applied an expanded system of scoring that incorporates that scoring approach and a battery of widely accepted criteria in ophthalmic practice. The objective criteria used objective provided a sound manner to quantitate the improvement in patient vision. In addition, our scoring system and its analysis identified several valuable features for future scoring improvement and surgical decision-making. The first observation to be made is that within the first year of follow up, the lion share of vision improvement occurred during the first trimester. The second notable, and somewhat counterintuitive observation was that the Postop score attained by the surgical intervention was not dependent on the degree of initial visual and ocular surface degradation These two features suggest that while the loss of function is primarily related to the catastrophic changes on the corneal epithelium/ supra stromal zone, stromal changes that affect vision occur simultaneously in a manner not directly correlated with the degree of changes at the corneal surface. Additionally, in the present study, other parametric tests were included such as OSDI, NEI-VFQ, subjective pain and visual loss scale in order to evaluate the outcomes. The meager improvement in pain sensation may relate to the latter concept. Although, the availability of a robustly documented set of scores will be very useful in the comparison across implementation of cell expansion, surgical methods and assessment of patient status over long periods in patients with LSCD due to ocular surface burns, it may not be applied to other causes of LSCD including aniridic keratopathy.

## Conclusions

Objective clinical metrics demonstrate that in patients with limbal stem cell deficiency, cultivated limbal epithelial cell transplantation improves vision and ocular surface health and subjective visual perceptions.

## Data Availability

All data generated or analyzed during this study are included in this published article [and its supplementary information files.

## Abbreviations

BCVA: Best-corrected visual acuity
OSS: Ocular surface score
NEI-VFQ 25: National Eye Institute Visual Function Questionnaire
OSDI: Ocular surface disease index
LSCD: Limbal stem cell deficiency
CLET: Transplantation of culture-expanded limbal epithelial cell
GLP: Good laboratory practices
LogMAR: Logarithm of the minimum angle of resolution
COASTL: Clinical outcome assessment in surgical trials
hAM: Human amniotic membrane
DMEM: Dulbecco Modified Minimal Essential Medium
